# Mental health and immigration

**DOI:** 10.1192/j.eurpsy.2021.856

**Published:** 2021-08-13

**Authors:** M.O. Solis, S. Jimenez Fernandez, M. ValverDe Barea

**Affiliations:** Jaén, Complejo Hospitalario Jaén, Jaén, Spain

**Keywords:** mental health, Ulysses Syndrome, Beck Inventory, immigration

## Abstract

**Introduction:**

Immigration entails uprooting and this is always a destabilizing event. It includes disorders in family life and a radical break with culture, values, among others. These events create a situation of uncertainty that exacerbates stress and anxiety.

**Objectives:**

Within this framework, we wanted to inquire about the state of mental health, and more specifically, about depression, in migrants.

**Methods:**

A cross-sectional study was carried out that includes 272 migrants from different countries of the world, during the months of August and September 2020, through an anonymous, voluntary and multiple response type online survey which included questions about sociodemographic aspects and the Beck Depression Inventory. The survey was published through social networks (Facebook and Whatapp) in migrant forums around the world, mostly Spanish-speaking, because the survey was published in Spanish.

**Results:**

Of the 276 immigrants surveyed, an average age of 36.63 is seen. The 30% are single. The 30.79% are unemployed. 0.72% attended an immigrant reception centre. 99.63% have Spanish as mother tongue. 33.33% emigrated alone. The results of Beck’s questionnaire, 28.98% have moderate/severe depression. Of the total respondents, 49.63% have been an immigrant for 1 to 5 years. The 85,14% were in a regular legal-inmigration situation.

**Conclusions:**

The immigrant population can be a group at risk for developing anxiety or depressive symptoms, especially when there is a situation of vulnerability and the necessary adaptation mechanisms for a satisfactory migration process cannot be guaranteed.

